# Time crystal dynamics in a weakly modulated stochastic time delayed system

**DOI:** 10.1038/s41598-022-08776-y

**Published:** 2022-03-22

**Authors:** Jordi Tiana-Alsina, Cristina Masoller

**Affiliations:** 1 Department de Física Aplicada, Facultat de Fisica, Universitat de Barcelona, Marti i Franques 1, 08028 Barcelona, Spain; 2 Departament de Fisica, Universitat Politecnica de Catalunya, Rambla Sant Nebridi 22, 08222 Terrassa, Barcelona Spain

**Keywords:** Nonlinear phenomena, Statistical physics

## Abstract

Time crystal oscillations in interacting, periodically driven many-particle systems are highly regular oscillations that persist for long periods of time, are robust to perturbations, and whose frequency differs from the frequency of the driving signal. Making use of underlying similarities of spatially-extended systems and time-delayed systems (TDSs), we present an experimental demonstration of time-crystal-like behavior in a stochastic, weakly modulated TDS. We consider a semiconductor laser near threshold with delayed feedback, whose output intensity shows abrupt spikes at irregular times. When the laser current is driven with a small-amplitude periodic signal we show that the interaction of delayed feedback and modulation can generate long-range regularity in the timing of the spikes, which lock to the modulation and, despite the presence of noise, remain in phase over thousands of modulation cycles. With pulsed modulation we find harmonic and subharmonic locking, while with sinusoidal modulation, we find only subharmonic locking, which is a characteristic feature of time-crystal behavior.

## Introduction

In many-particle systems, when the translation symmetry in space (in time) is spontaneously broken, the result is a space (time) crystal. Time-crystal states are characterized by highly regular oscillations that are stable over very long times, are robust under perturbations (“rigidity”) and break time-translation symmetry^1,2^. Time-crystal states were originally introduced for many-particle systems in equilibrium^3^, and after the possibility of such states in equilibrium systems was ruled out^4,5^, they were investigated in non-equilibrium systems under a periodic drive^6^. In this case, the periodic forcing defines the discrete time translation symmetry and this symmetry is broken in the time crystal phase, where ’discrete time-crystals’ states occur^7–9^. In these states the system’s variable displays sub-harmonic oscillations that are rigidly locked to the driving signal. In recent years, time-crystal behavior has been studied in a wide range of systems^10–19^.

A key requirement for observing discrete time-crystal dynamics is that the system’s oscillations have long-term regularity that results from the collective synchronization of many interacting degrees of freedom. This excludes period-doubling oscillations in low-dimensional dynamical systems, and it also excludes oscillations in mode-locked lasers, which arise due to interactions of many modes but which lack long-term regularity because, due to noise, they do not remain in phase for long times^1^.

Here we address the following questions: Can a feedback loop counteract the effect of noise? Can a delayed feedback loop generate long-term order? Can we find discrete time-crystal-like behavior in periodically driven stochastic systems with feedback loops?

Time-delayed systems (TDSs) with feedback loops, governed by equations of the form $du(t)/dt = f (u(t),t)+Ku(t-\tau )$, have an infinite phase space because the initial condition is the function *u*(*t*) defined in [-$\tau$,0]^20,21^. In this type of TDS, when the feedback delay time, $\tau$, is longer than the internal characteristic time-scale of system, analogies have been found with the dynamics of one-dimensional spatially extended systems (1D SESs) governed by equations of the form $\partial u(x,t)/\partial t = f(u,x,t) + D \partial ^2 u/\partial x^2$ with *x*(*t*) in [0, *L*]. Specifically, in the TDS, $\tau$ plays the role of the size, *L*, in a 1D SES. When $\tau$ is long enough, using the so-called space-time representation^22,23^, complex spatio-temporal phenomena has been found, such as pattern formation and propagation of defects and localized structures, analogous to that occurring in 1D SESs^24–33^. These analogies make periodically driven stochastic TDSs promising test benches for finding non-trivial time-crystal-like oscillations.

A semiconductor laser with optical feedback is a well-known TDS, where the delay is due to the finite propagation time of the feedback light. Optical feedback from a distant reflector introduces a set of new modes (the so-called external cavity modes) and generates a complex intensity dynamics^34^ that, when viewed using the space-time representation, reveals spatio-temporal patterns and defects typical of 1D SES^35^.

Near threshold and for appropriated feedback parameters the laser output intensity displays spikes that occur at irregular times. A typical intensity time series is shown in Fig. 1a and a video of the spiking dynamics can be found in^36^. In this regime the laser dynamics is strongly influenced by noise: simulations of the well-known Lang-Kobayashi model^37^ indicate that when a noise term is not included in the model, the spikes are periodic or are transient^38–40^. When the laser current is modulated with a small-amplitude periodic signal, the spikes tend to occur at intervals of time that are integer multiples of the period of the modulation^41–49^.

**Figure 1:**
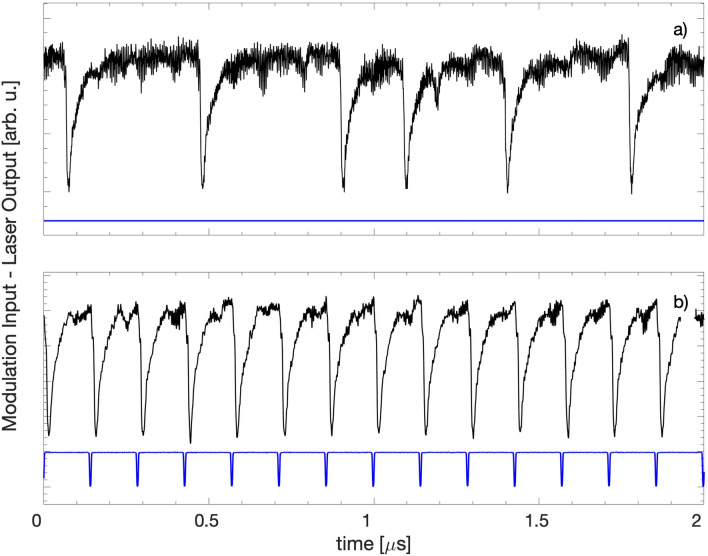
Laser intensity without modulation (**a**) and with pulsed modulation (**b**). In both panels the dc value of the laser current is $I_{dc}$=26 mA. In (**b**) the modulation amplitude is 0.631 mA ($\sim$2.4% of $I_{dc}$) and the frequency is $f_{mod}=7$ MHz. The pulsed modulation generates periodic spikes that are harmonically locked to the modulation; however, in between the spikes, the intensity fluctuations are irregular.

We have recently shown^50^ that a small-amplitude pulsed modulation (less than 2.5% of the dc value of the laser current) generates regular 1:1 locked spikes. A typical intensity time series is shown in Fig. 1b (details of the experimental setup and parameters can be found in^50^). In Fig. 1b we see that the spikes are periodic but in between the spikes the intensity shows irregular fluctuations.

## Results

To determine whether the laser with delayed feedback and weak modulation can display non-trivial discrete time-crystal-like dynamics, we begin by analyzing how regular the spike timing is, and how the spike timing regularity is affected by the waveform and by the parameters of the driving signal. The experimental dataset (described in^50^) consists of time series of the laser output intensity recorded over an interval of 5 ms. We analyze the effects of sinusoidal and pulsed modulation, varying the modulation frequency, $f_{mod}$, and the dc value of the laser current, $I_{dc}$, keeping constant the modulation amplitude (0.631 mA). The intensity time traces contain between 9000 spikes (for low $I_{dc}$ and $f_{mod}$) and 120000 spikes (for high $I_{dc}$ and *f*).

**Figure 2:**
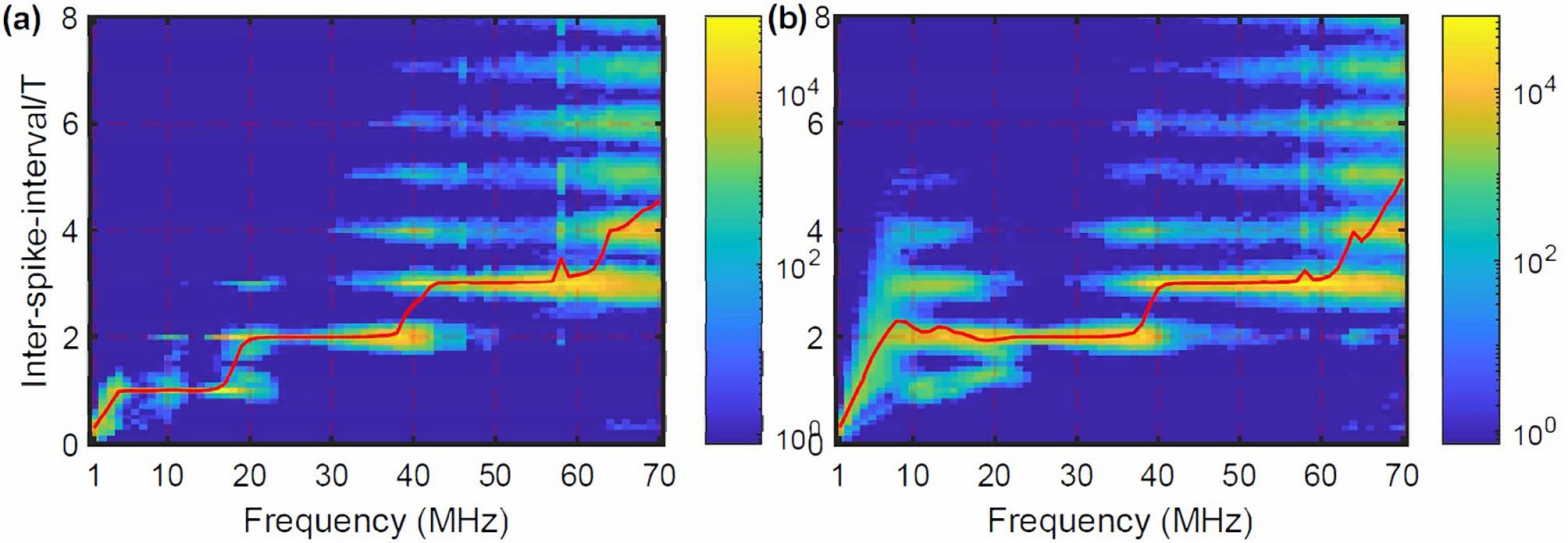
Inter-spike-interval (ISI) distribution in log color code for pulsed (**a**) and for sinusoidal (**b**) modulation, as a function of the modulation frequency; other parameters are as in Fig. 1b. The solid line indicates the mean ISI normalized to the modulation period. The plateau at low frequencies for pulsed modulation [panel (**a**)] where $\left<\text {ISI}\right>/T_{{mod}}=1$ is not seen in panel (**b**), where the modulation is sinusoidal.

In Fig. 2 we compare the distribution of time intervals between consecutive spikes (inter-spike-intervals, ISIs) generated by pulsed modulation (left) and by sinusoidal modulation (right), as a function of the modulation frequency. The vertical axis displays the ISI normalized to the modulation period, $T_{{mod}}$, and the distribution of ISI values in shown in log color code. We see that for particular modulation frequencies the ISI distribution is narrow, and the ISIs are multiples of $T_{{mod}}$. For the pulsed waveform, Fig. 2a, there are three “plateaus” where $\text {ISI}/T_{{mod}}$=1 or 2 or 3 (a spike is emitted every one, two or three modulation cycles), but for the sinusoidal waveform, Fig. 2b, there are only two plateaus where $\text {ISI}/T_{{mod}}$=2 or 3.

Therefore, pulsed modulation generates harmonic and subharmonic locking, while sinusoidal modulation, only subharmonic locking. In Fig. 2b we note that for $f_{mod}\approx 3$ MHz, $\left<\text {ISI}\right>/T_{{mod}}\approx 1$, i.e., the sinusoidal waveform generates, *on average*, a spike per modulation cycle; however, there is no locking because the ISI distribution is broad and there is no plateau, i.e., there is no interval of frequencies in which $\left<\text {ISI}\right>/T_{{mod}}\approx 1$. These observations are consistent with previous experiments using small-amplitude sinusoidal modulation^47,48^, where we found subharmonic locking but not 1:1 locking.

The lack of 1:1 locking generated by small-amplitude sinusoidal modulation contrasts with the dynamics of low-dimensional (non-delayed) dynamical systems, where small-amplitude sinusoidal modulation can generate harmonic locking^51,52^. It also differs from the stochastic resonance phenomenon^53^ that has been observed in many noisy oscillators, including in a similar laser system^45^, whose characteristic feature is a peak in the ISI distribution at $\text {ISI}/T_{{mod}}$=1, whose strength is maximum for appropriated parameters.

With sinusoidal current modulation harmonic locking has been observed experimentally; however, under large-amplitude modulation ($\sim$14% and 20% of $I_{dc}$ in^42^ and^49^ respectively). With large-amplitude modulation the feedback-induced spikes are a perturbation of a sinusoidal-like oscillation. To shed light on this situation we have carried out simulations of the Lang-Kobayashi (LK) model^37^ and found a good agreement with the observations: large-amplitude sinusoidal current modulation produced harmonic locking, while small-amplitude modulation produced sub-harmonically locked spikes^54^.

To precisely quantify the regularity of the spike timing we calculate the Fano factor^55^, *F*, which is a well-known measure of the variability of sequences of events^56^, described in Sec. *Methods*.

**Figure 3:**
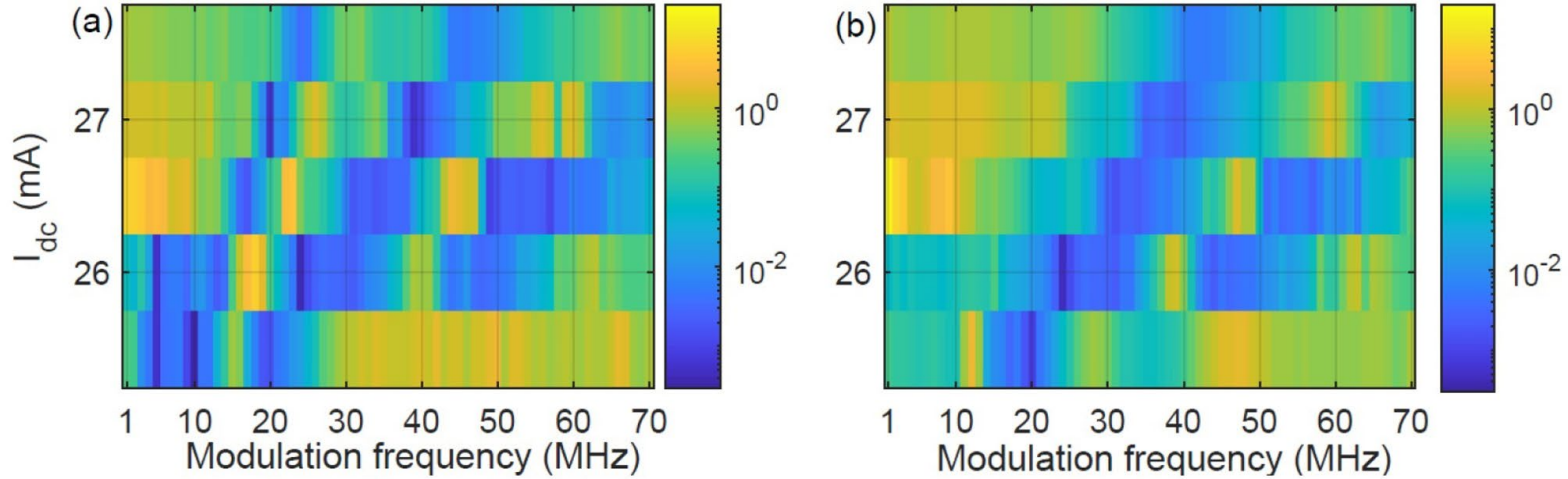
Fano factor (in log color code) versus the modulation frequency and the dc value of the laser current, $I_{dc}$, for pulsed (**a**) and for sinusoidal (**b**) modulation. The blue region located at low frequencies in panel (**a**), where the spikes are 1:1 locked to the modulation is not seen in panel (**b**).

Figure 3 displays the Fano factor (in log color code) of sequences of spikes recorded for different $I_{dc}$ and $f_{mod}$. In panel (a) the modulation is pulsed, while in (b), it is sinusoidal. In both panels we see parameter regions where $F<10^{-2}$ (dark-blue) and regions where $F>1$ (yellow). We remark that $F<1$ ($F>1$) indicates that, in the time scale of $5~\upmu$s, the sequence of counts is more (less) regular than a sequence of events generated by a HPP process.

Comparing with the ISI distributions shown in Fig. 2 for $I_{dc}=26$ mA, we see (as expected) that the blue regions in Fig. 3 correspond to the “plateaus” in Fig. 2 where the ISI distribution is narrow and thus, the spike timing is regular. In Fig. 3 we see that for the pulsed signal there are three blue regions, while for the sinusoidal signal, only two, confirming that 1:1 locking is not generated by small-amplitude sinusoidal modulation.

We interpret the different response of the laser to low-frequency modulation, pulsed or sinusoidal, as due to two reasons. First, the pulsed signal produces small but abrupt variations of the pump current that precisely define the timing of the spikes (the current changes at instants of time, producing spikes at those times); in contrast, the sinusoidal signal varies the laser current gradually, and this variation is unable to precisely define the spike timing. Second, the pulsed signal decreases the current for only short time intervals, while the sinusoidal signal decreases the current during longer intervals, which may lead to a stronger influence of noise.

**Figure 4:**
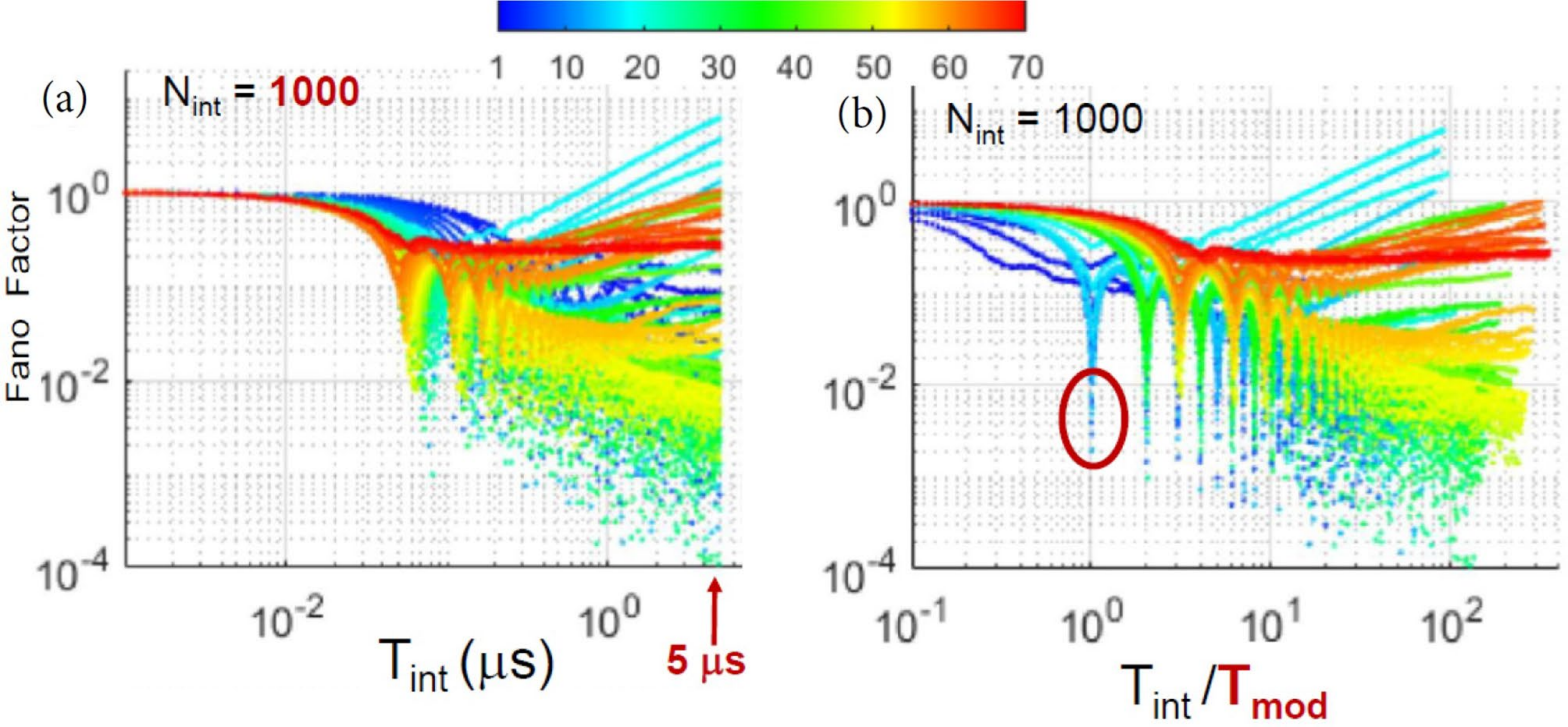
(**a**) Fano factor versus the duration of the counting interval, $T_{\text {int}}$. The modulation is pulsed, $I_{dc}=26$ mA, and the color code indicates the modulation frequency in MHz. (**b**) *F* versus $T_{\text {int}}$ normalized to the period of the modulation, $T_{{mod}}$ (i.e., *F* vs. the number of modulation periods contained in the counting interval). *F* decreases sharply when $T_{\text {int}}/T_{{mod}}=n$ with $n\ge 1$.

**Figure 5:**
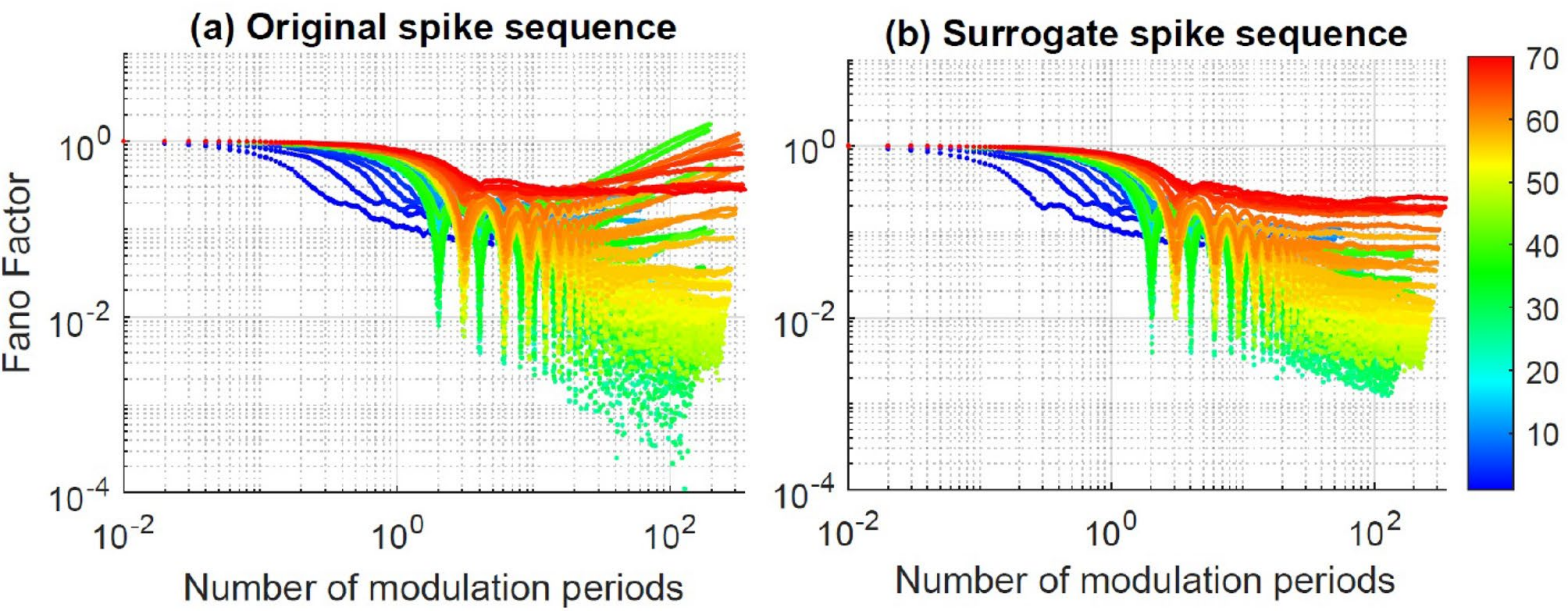
(**a**) Fano factor vs. the number of modulation periods contained in the counting interval, $T_{\text {int}}/T_{{mod}}$. The experimental parameters are as in Fig. 4b, but the modulation waveform is sinusoidal. (**b**) *F* is computed from the shuffled sequence of spikes. In both panels, *F* dips when $T_{\text {int}}/T_{{mod}}=n$ with $n\ge 2$.

To analyze the time scale of the spike timing regularity, we study how *F* depends on the duration of the counting interval, $T_{\text {int}}$, and on the modulation frequency, $f_{mod}$ (in color code). Figure 4 displays results for pulsed modulation, when $I_{dc}=26$ mA. Panel (a) displays *F* vs. $T_{\text {int}}$, and we see that, as expected, $F\rightarrow 1$ when $T_{\text {int}}$ is short enough, for all $f_{mod}$. For the longest $T_{\text {int}}$ (5 $\upmu$s), *F* varies in the range $10^{-4}-10^{1}$, depending on $f_{mod}$. After re-scaling the horizontal axis to the period of the signal, panel (b), we see that, for some modulation frequencies, *F* dips sharply when $T_{\text {int}}/T_{\text {mod}}=n$ with $n=1,2,3,\dots$. These minima reveal that, for frequencies that produce locked spikes, the sequence of counts is very regular when the counting interval contains an integer number of periods. In contrast, when the modulation is sinusoidal, Fig. 5a, the first dip occurs for $T_{\text {int}}/T_{\text {mod}}=2$ and no modulation frequency produces a dip at $T_{\text {int}}/T_{\text {mod}}=1$, confirming that a small-amplitude sinusoidal modulation does not generate harmonic locking.

In Figs. 4 and 5a we also note that for some frequencies *F* grows with $T_{\text {int}}$ as a power law. This behavior reveals spike clustering (bursts of spikes) and has been observed in sequences of events, in different fields^56–58^.

To determine whether the *F* values are due, in part, to “dynamic” properties of the spike sequence (the presence of temporal correlations between the spike times), or they are only due to “static” properties (the shape of the ISI distribution) we shuffled the sequence of ISIs, recalculated the spike times^59^, re-calculated *F*, and compared the *F* values of the original and shuffled spike sequence. The presence of long-range order in the timing of the spikes is detected by *F* values that are significantly higher or lower in the original spike sequence than in the shuffled one. The high or low variability of the sequence of counts reveals the presence of temporal correlations between spikes, which are washed out when we randomly shuffle the values in the ISI sequence.

The Fano factor of the shuffled spikes is displayed in Fig. 5b, where we see that the dips are less pronounced with respect to those in the original spike sequence, Fig. 5a, and we also note that the power law grow disappears in the shuffled data. These differences reveal the presence, in the original spike sequence, of long-range correlations in the spike timing, which are removed after shuffling the ISIs.

The presence of long-range correlations in the spike timing is intriguing at first sight, because near threshold the spiking dynamics of the un-modulated laser is irregular, and we modulate the laser with a small amplitude signal (for $I_{dc}=26$ mA the modulation produces a variation of the laser current <3%). However, the delayed feedback introduces a degree of periodicity, and particular combinations of the feedback and modulation parameters can induce spikes that rigidly lock to the modulation with high timing regularity.

**Figure 6:**
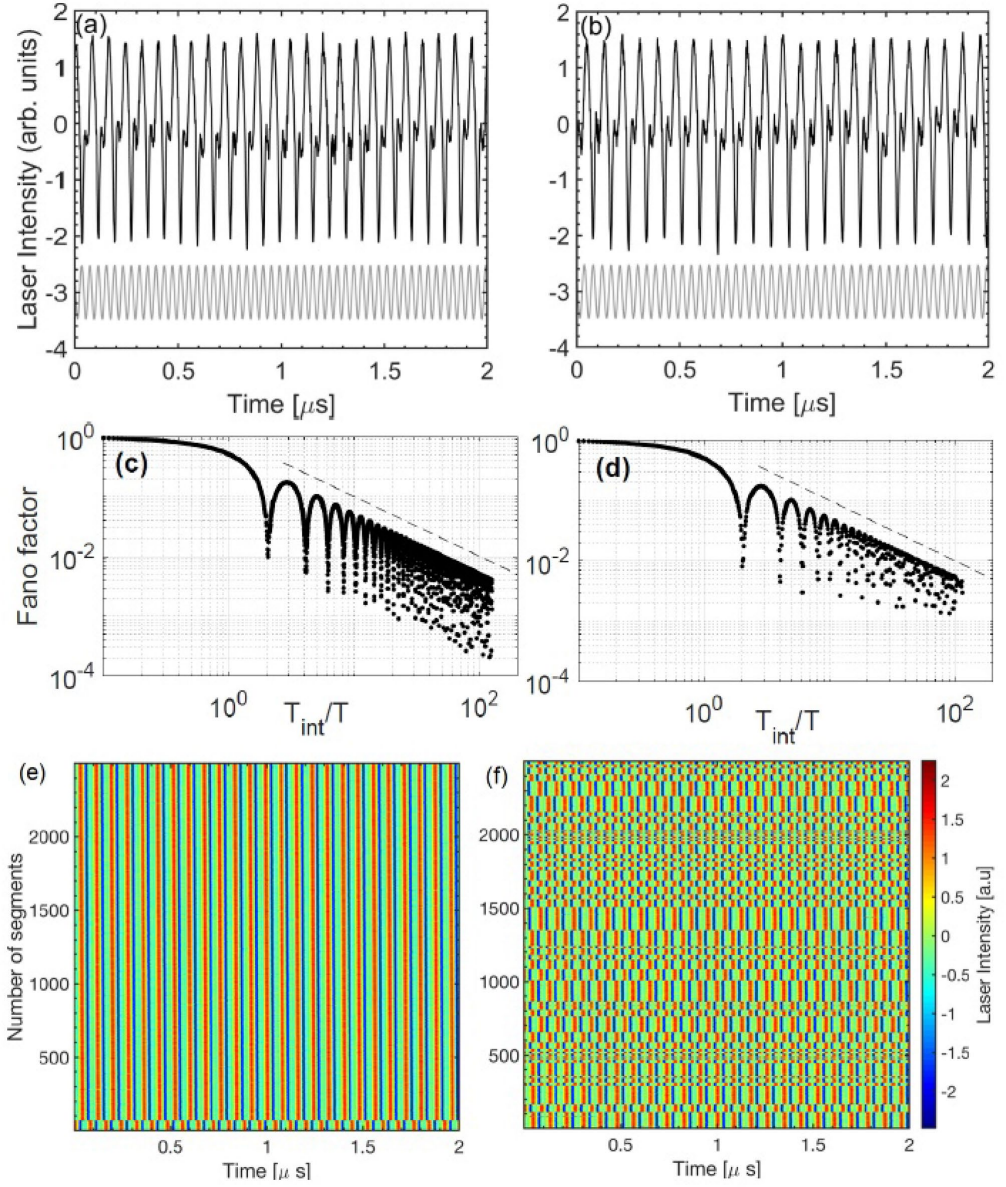
Comparison of different long-term locked behaviors when $I_{dc}= 26$ mA and the frequency of the sinusoidal modulation is 25 MHz (**a**, **c**, **e**), 23 MHz (**b**, **d**, **f**). (**a**), (**b**) Short segment of the intensity time series; (**c**), (**d**) *F* versus $T_{\text {int}}/T$; (**e**), (**f**) spatio-temporal representation of the intensity time series: all the intensity values $\{I_i, i= 1\dots 10^7\}$, are plotted in color code versus *t* and *n* such that $i= n \Delta T + t$ and $\Delta T$=2 $\upmu$s.

For most frequencies that generate locked spikes, the dips for $T_{\text {int}}=nT_{\text {mod}}$ gradually disappear when $T_{\text {int}}$ increases, revealing that the spike timing regularity does not persist over long intervals, i.e., the spikes do not remain in phase. However, for particular modulation frequencies *F* decreases with $T_{\text {int}}$ as $T_{\text {int}}^{-1}$, and the dips for $T_{\text {int}}=n T_{\text {mod}}$ persist for large *n*. Figure 6 shows two examples of locked spikes, with and without long-range regularity, generated by sinusoidal modulation with frequency 25 MHz and 23 MHz, respectively. As shown in panels (a) and (b), differences can not be distinguished when the intensity dynamics is inspected in a short time interval. In both panels we see that the spikes are periodic but the intensity dynamics is not, because in between spikes, the oscillations are irregular (a similar behavior -regular spikes and irregular oscillations in between spikes- was observed in Fig. 1b). However, if we examine the dynamics over longer time intervals, the spike timing regularity disappears when $f_{mod}=23$ MHz, while it persists when $f_{mod}=25$ MHz. This difference is seen in the plots of the Fano factor, panels (c) and (d), and also, in the space-time representation of the intensity time series, (e) and (f).

**Figure 7:**
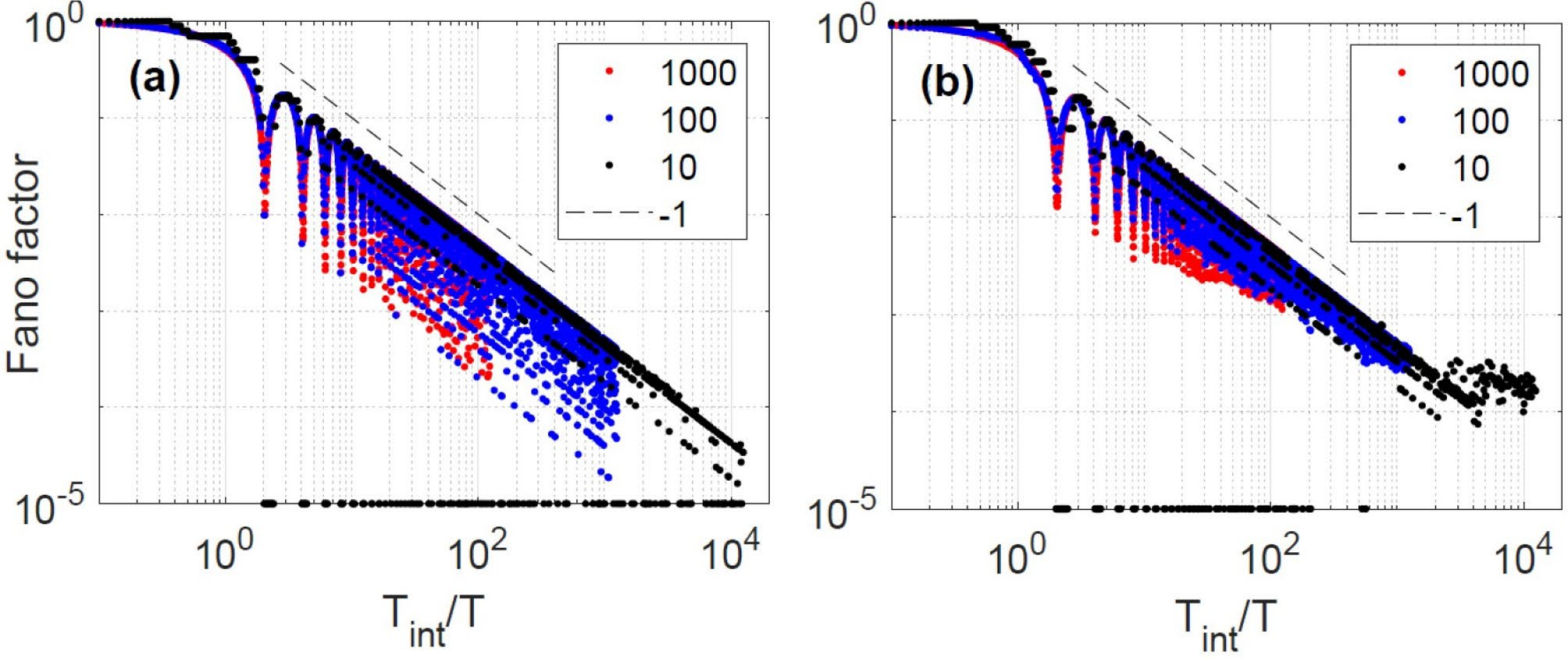
Fano factor of the sequence of spikes generated by sinusoidal modulation with $f_{mod}=25$ MHz and $I_{dc}=26$ mA. In (**a**) *F* is calculated from the original spike sequence and in (**b**) from the shuffled one, using $N_{\text {int}}=$10, 100 or 1000 spike counting intervals. To represent $F=0$ in log scale, we set it to $F=10^{-5}$.

To demonstrate that the regularity of the spike timing generated by sinusoidal modulation with frequency 25 MHz is indeed very long range, we calculate the Fano factor considering longer counting intervals (so far we divided the intensity time series in 1000 intervals of 5 $\upmu$s each). To increase the length of the intervals, we need to decrease the number of intervals. The results are shown in Fig. 7a, where we see that *F* continues decreasing as $T_{\text {int}}^{-1}$, even when we calculate *F* using intervals of 500 $\upmu$s each (each interval contains 12,500 modulation cycles). In the shuffled spike sequence, Fig. 7b, we see that for large $T_{\text {int}}$ the decrease of *F* saturates and the dips disappear. This confirms a long-range timing regularity in the original spike sequence, which is removed in the shuffled spike sequence, where *F* values are low just because of the narrow shape of the ISI distribution. This long-range suppression of fluctuations in the timing of the spikes on time scales that contain thousands of modulation cycles, together with the aperiodic nature of the oscillations in between spikes and the lack of 1:1 locking, allows us to interpret this spiking behavior as a non-trivial form of time-crystal dynamics.

## Discussion

Summarizing, we have found that near threshold a weak modulation of the laser current induces, for particular modulation conditions, long-range regularity in the timing of the spikes, as revealed by Fano factors that decrease with the length of the spike counting interval as $T_{\text {int}}^{-1}$. With pulsed modulation we found harmonic and subharmonically locked spikes, but with sinusoidal modulation, we found only subharmonic locking, which is a common feature with time-crystal behavior. We have interpreted the difference in the laser response to pulsed and sinusoidal modulation as due to (1) the existence of the lasing threshold and (2) the fact that harmonic locking occurs for low modulation frequencies. In these conditions the pulsed modulation decreases sharply the laser current, but only for short time intervals, inducing spikes with well-defined periodicity. In contrast, the sinusoidal modulation decreases the current gradually, and this smooth variation brings the laser current close to threshold for longer intervals, allowing for a larger influence of noise and preventing the generation of harmonically locked spikes. Therefore, we have found, in a stochastic dynamical system, discrete time-crystal-like behavior generated by the interplay of periodic modulation and delayed feedback.

Regarding the level of noise, compared to other types of lasers, semiconductor lasers are more affected by quantum spontaneous emission because of the phase-amplitude coupling of the optical field (due to the variation of the semiconductor refractive index with the carrier population, an effect that is phenomenological modeled by Henry’s alpha factor^38,60^). Other sources of noise include electric, thermal and mechanical fluctuations. We have not measured the level of noise in our system, but we have performed extensive simulations of the dynamics of the laser with optical feeedback and sinusoidal current modulation using the LK model^37^ and found a very good agreement experiments-simulations, when the model includes an stochastic term that takes into account spontaneous emission, and disregards other sources of noise^61,62^.

Our findings open a new path for studying the emergence of long range regularity in disordered systems, exploiting the analogy between time-delayed systems and spatially-extended ones. This analogy can be extended to the transverse section of a broad area laser, where, under optical injection, localized structures (that can be regarded as spikes) are created and move as the result of the combined action of nonlinearity, diffraction, carrier diffusion and delayed feedback^63,64^.

The harmonically locked spiking behavior with long-range regularity generated by pulsed modulation may be consider analogous, in the temporal domain, to hyper-uniform states in multi-particle systems, which are characterized by long length scale suppression of density fluctuations^65^. Moreover, because delayed feedback is a popular control technique with multiple applications, we expect that our observations will motivate studies to generate, characterize, and exploit the highly regular oscillations that can be generated by the interplay of nonlinearity, feedback and modulation.

## Methods

To compute the Fano Factor we first divide the intensity time trace in $N_{\text {int}}$ non-superposing segments of duration $T_{\text {int}}$ and count the spikes in each segment; then, we calculate $F=\sigma ^2(N_i)/\left<N_i\right>$, where $\sigma ^2(N_i)$ and $\left<N_i\right>$ are the variance and the mean of the sequence of counts, $\{N_i,i=1\dots N_{\text {int}}\}$. *F* is a function of $T_{\text {int}}$; if $T_{\text {int}}$ is very small, $F=1$ because the sequence of counts is a sequence of 0s and 1s, while in time scales over which the spikes are regular, the variance of $\{N_i\}$ is small and *F* takes low values. Unless specifically stated, to calculate the Fano factor we divide the intensity time-series in $N_{\text {int}} = 1000$ non-overlapping segments of $T_{\text {int}}=5~\upmu$s each.

If the spikes are generated by a fully random, homogeneous Poisson point (HPP) process, the probability that *N* spikes occur in an interval $T_{\text {int}}$ is $p(N,T_{\text {int}})=(\lambda T_{\text {int}})^N \exp (-\lambda T_{\text {int}})/N!$ where $\lambda$ is the average number of spikes per unit time (i.e., the spike rate). For this distribution $\sigma ^2(N_i) = \left<N_i\right> = \lambda T_{\text {int}}$ and thus, $F=1$ $\forall$ $T_{\text {int}}$^56^.

## Data Availability

The experimental sequences of inter-spike-intervals are available here 10.5281/zenodo.5913506.
